# Development and characterization of high-throughput serological assays to measure magnitude and functional immune response against *S.* Paratyphi A in human samples

**DOI:** 10.3389/fimmu.2024.1443137

**Published:** 2024-10-30

**Authors:** Martina Carducci, Luisa Massai, Elisa Lari, Bianca Semplici, Silvia Grappi, Noshi Maria, Elizabeth Jones, Valentino Conti, Pietro Piu, Francesco Berlanda Scorza, Miren Iturriza-Gómara, Emanuele Montomoli, Andrew J. Pollard, Simona Rondini, Omar Rossi

**Affiliations:** ^1^ GlaxoSmithKline (GSK) Vaccines Institute for Global Health (GVGH) S.r.l., Siena, Italy; ^2^ VisMederi S.r.l., Siena, Italy; ^3^ Oxford Vaccine Group, Department of Paediatrics, University of Oxford, and the National Institute for Health and Care Research (NIHR) Oxford Biomedical Research Centre, Oxford, United Kingdom; ^4^ Department of Molecular and Developmental Medicine, University of Siena, Siena, Italy

**Keywords:** enzyme-linked immunosorbent assay (ELISA), serum bactericidal assay (SBA), human sera, functional assay, antibodies, vaccines, enteric fever

## Abstract

Typhoid and Paratyphoid fever cause a global health burden, especially for the children of Southern Asia. The impact of the disease is further exacerbated by the dramatic increase of antimicrobial resistance. While vaccines against *Salmonella* Typhi have been developed and successfully introduced, an effective vaccine targeting *S.* Paratyphi A is still lacking. Several efforts are currently ongoing to develop vaccines targeting both *S.* Typhi and *S.* Paratyphi A. In order to analyze the immune response induced by vaccination and in sero-epidemiological studies, easy to perform and high throughput immunoassays are needed. Here we present the setup and characterization of a customized ELISA assay and of a luminescent-based serum bactericidal assay (L-SBA) to measure the quantity of *S.* Paratyphi O antigen specific antibodies and their functional activity against *S.* Paratyphi A. Robust quality control criteria have been put in place both for ELISA and SBA and assays have been fully characterized in terms of quantitation limit, limit of blanks, specificity, linearity and precision. Assays are being employed to analyze samples from clinical trials, enabling the assessment of immunogenicity during clinical vaccine development.

## 1 Introduction

Despite improved access to clean water and adequate sanitation in the last decade, enteric fever remains a major cause of morbidity and death, with billions of people likely exposed to *Salmonella* enterica pathogens causing typhoid and paratyphoid fever (1). In 2019, there were at least 13 million cases of enteric fever globally, 28% of which caused by *Salmonella* Paratyphi A (2). Increasing incidence of *S.* Paratyphi A has been registered in some parts of Asia, with up to 35% of enteric fever cases in India and Nepal and >60% in China attributable to *S.* Paratyphi A. As for *S.* Typhi, multidrug resistant strains of *S.* Paratyphi A are increasingly being detected (3).

Vaccines that can effectively prevent *S.* Typhi in young children have been developed (4–7) and in 2018 WHO recommended their adoption in endemic countries. Since then, three typhoid conjugate vaccines (TCV) have obtained WHO-prequalification and two of them have been successfully introduced into routine immunization with funding from GAVI (Global Alliance for Vaccines and Immunization).

However, no vaccines exist to control paratyphoid fever and their development is delayed by lack of immunological correlates of protection and of suitable animal models of infection. Recently, an *S.* Paratyphi A controlled human infection challenge model (CHIM) has been developed (8), and it will possibly inform on immunological readouts associated with infection and protection. Another important factor that will facilitate the development of an effective vaccine will be the generation of international reference reagents to better describe and harmonize the immunological response against *S.* Paratyphi A, during natural infection, or candidate vaccine immunization.

To extend the coverage of TCV and achieve protection also against *S.* Paratyphi A, various efforts are currently ongoing to develop a bivalent typhoid-paratyphoid vaccine (4, 9–11). More specifically, our approach consists in combining a Vi-CRM197 conjugate (Typhibev, manufactured by Biological E) with another glycoconjugate vaccine, composed by the *S.* Paratyphi A serovar-specific O-antigen (O:2) conjugated to CRM197 (O:2-CRM197).

In preparation to analyze samples from clinical trials, we developed serological assays to determine the quantity and the functionality of the induced antibodies. Here we present the development and characterization of an ELISA to quantify anti-O:2 IgG antibodies, and of a high throughput serum bactericidal luminescence based assay (L-SBA) to assess the ability of sera from immunized subjects to kill a *S.* Paratyphi A clinical strain.

This newly developed ELISA assay for *S.* Paratyhi A is based on standardized assays already in place to detect anti-O-antigen IgG of other serovars of *Salmonella* enterica (*S.* Typhimurium and *S.* Enteritidis) (12) and has been characterized based on ICH Guidelines (13), in terms of accuracy of standard curve, dilutional linearity, repeatability, intermediate precision and specificity.

As for ELISA assay, L-SBA has been setup, based on previously described (14) by adapting a qualified assay for Shigella, currently used to evaluate a vaccine candidate in clinical development (15). The assay parameters evaluated in this case are intermediate precision and repeatability, limit of quantitation and detection, and linearity.

In addition, we employed both assays to test clinical samples derived from a *S.* Paratyphi A CHlM (8, 16) to test the performance of the assays with samples from individual subjects and to compare results between laboratories, using different methods. Comparing results within laboratories and across studies is particularly relevant in the absence of an international standard.

## 2 Material and methods

### 2.1 Enzyme-linked immunosorbent assay

Anti-*S.* Paratyphi A OAg specific total IgG were measured in sera samples using O:2 as coating antigens adapting protocol as described in (12). In brief NuncMaxisorp 96-well round bottom (Nunc) plates were coated with O:2 antigen [extracted from ED199 strain and fully characterized for O-antigen content, O-antigen size, sugar composition, O-acetylation level and impurities (17)] at a final concentration of 2 µg/mL in Carbonate buffer (pH 9.6) and incubated at 4°C overnight; then coating solution was aspirated (without wash) and blocked with 5% PBS milk for 1 h at 25°C; after three washes with PBS-Tween 0.05%, primary antibodies, diluted in 5% PBS milk, were added and incubated for 2 h at 25°C. Plates were then washed 3 times with PBS-Tween 0.05% and incubated for 1 h at 25°C with Alkaline Phosphatases conjugated secondary antibodies diluted 1:5000 in 0.1% BSA PBS-Tween. Then plates were washed again 3 times with PBS-Tween and p-Nitrophenyl phosphate substrate (Sigma-fast, Sigma-Aldrich, Massachusetts, United States) was added and incubated for 1 h at 25°C. Absorbances at 405 and 490 nm were acquired using an automatic plate reader (Biotek). The GVGH standard ELISA uses the following layout: sera were assayed at different dilutions: 1:100, 1:4,000 and, if necessary, 1:160,000. Each serum dilution was prepared as single sample that was assayed in triplicate with triplicates on different ELISA plates. Up to 70 different test sera can be assayed on 96-well plates in one ELISA triplicate. Low Control (LC) serum and High Control (HC) serum were also added at appropriate dilution. Ten dilutions points (2-fold steps apart) of anti-antigen specific human standard serum at defined ELISA Units/mL (EU/mL) were assayed in duplicate on each ELISA plate, together with four blank wells used as negative control on the ELISA plate and as additional standard points. One ELISA unit is defined as the reciprocal of the dilution of the standard serum that gives an absorbance value equal to 1 in a standard assay.

### 2.2 Luminescent-based serum bactericidal assay

The high-throughput L-SBA is an antibody dependent and complement mediated killing assay (18). The level of luminescence detected is directly proportional to the number of living bacteria present in the wells which is inversely proportional to the level of functional antibodies present in the serum. Results of the assay are expressed as the IC50 (the dilution of sera able to kill half of the bacteria present in the assay), thus representing the SBA titer of the sera.

The used L-SBA protocol was described in (14), in brief *Salmonella* Paratyphi A ED199 strain was stored frozen at −80°C in 20% glycerol stocks and grown at 37°C in Luria Bertani (LB) medium overnight culture the day before the experiment. The bacterial suspensions were then diluted in fresh LB to start a new liquid culture from an optical density at 600 nm (OD600) of 0.05 and incubated at 37°C with 180 rpm agitation in an orbital shaker, until they reached 0.22 +/- 0.02 OD600. Sera and bacteria were diluted in LB medium and 20% of baby rabbit (3- to 4-week-old) serum (BRC-Cederlane) is added in the L-SBA reaction and incubated for 3 hours at 37°C. Then bacteria were pelleted by centrifuging the plates at 4,000 g for 10 minutes, supernatant is discarded and pellet was resuspended in PBS and mixed 1:1 V:V with BacTiterGlo (Promega) substrate. Luminescence signal was acquired by a luminometer after 5 minutes.

### 2.3 Serum samples and ethical statements

A *Salmonella* Paratyphi A primary standard serum and five positive control sera at different reactivity have been generated by screening for anti-O:2 specific response after natural exposure, sera from clinical trial aimed to assess safety and immunogenicity of Vi-CRM_197_ vaccine Against *S.* Typhi conducted in India and Pakistan where *S.* Paratyphi A is endemic (4) (NCT01229176). Pooled sera were aliquoted and stored at -80°C until use.

A subset of 24 serum samples belonging to two CHIM studies have been provided by University of Oxford to compare IgG titers retrieved by GVGH ELISA with original analysis (8, 16). Subjects underwent oral challenge with 0.5-1 or 1-5 x 10^3^ Colony Forming Units (CFU) (8) or re-challenge (volunteers previously-exposed to *Salmonella* Paratyphi A in earlier CHIM studies) with 10^3^ CFU (16). Written informed consent was obtained before enrollment from all subjects and the trial was designed and conducted in accordance with the Good Clinical Practice Guidelines and the Declaration of Helsinki.

### 2.4 Sample preparation to assess precision, linearity, limit of quantification, and specificity

Reference serum and controls were used as described below to assess different assays parameters such as precision, linearity, specificity and limits of detection and quantification.

#### 2.4.1 Samples used to assess standard curve accuracy of ELISA assay

*S.* Paratyphi A human standard serum has been used to prepare 24 standard curves, each composed of ten calibrators starting from 10 EU/mL and 2-fold diluted and 2 blanks.

#### 2.4.2 Samples used to assess precision, and the lower and upper limit of quantification

*S.* Paratyphi A standard serum and 5 control samples with different anti-O:2 titers were assayed by two operators, in single independently handled replicates on each plate, on three different days (18 and 72, for ELISA and L-SBA (only standard serum), respectively, measurements in total for each individual serum).

#### 2.4.3 Samples to assess linearity

*S.* Paratyphi A Standard Serum was assayed in ELISA assay at 9 independent dilutions (from neat to 1:256 diluted, 2 fold apart in negative matrix (Human IgG depleted serum, Molecular Innovations cod. HPLA-SER-GF)) prior to probing it as a sample in the assay; each dilution was prepared for 2 times independently. *S.* Paratyphi A Standard serum serum was pre-diluted in PBS (neat, and then at other 5 dilutions, 1.5-fold apart) before being probed against *Salmonella* Paratyphi A in L-SBA.

Samples were incubated O/N at 4°C prior being tested in ELISA and L-SBA.

#### 2.4.4 Sample to assess specificity

*S.* Paratyphi A Standard serum was incubated with O:2 or *S.* Paratyphi A GMMA (19) to prepare inhibited samples for ELISA and L-SBA, respectively.

To establish the concentration of homologous antigen able to inhibit of >80% of EU/mL of the inhibited sample in ELISA, reference standard was incubated O/N at 4°C with an equal volume of homologous competitor at the final concentrations of 250, 50, 20, 5, 1 µg/mL in 5% milk in 1x PBS prior to being tested; reference standard was also preincubated O/N at 4°C with an equal volume of 5% milk in 1x PBS alone to represent the “control” uninhibited sample. To assess the homologous and heterologous specificity in ELISA assay, reference standard was inhibited with homologous or heterologous antigens at the same concentration of homologous antigen able to inhibit of >80% of EU/mL of the non inhibited sample in ELISA as established in the setup experiment (250 µg/mL). For heterologous specificity Vi from Citrobacter (polysaccharide heterologous equivalent to the one present in the same species – *S.* Typhi – and other antigen in the bivalent vaccine) and Shigella flexneri 1b OAg (heterologous from a different species) were tested; internal controls for this experiment were represented by a sample preincubated with an equal volume of 5% milk in 1x PBS alone (undepleted) and a sample preincubated with homologous *S.* Paratyphi A OAg (homologous).

To assess homologous and heterologous specificity in L-SBA a similar experiment to the one described for the ELISA has been performed, initially by testing different concentrations of *S.* Paratyphi A GMMA to deplete every possible bactericidal activity to the reference serum (anti-OAg and/or anti-protein antibodies). To assess the heterologous specificity sample of reference standard incubated with a final concentration of 40 µg/mL of homologous (*S.* Paratyphi A GMMA) and heterologous Vi from Citrobacter (polysaccharide heterologous equivalent to the one present in the same species – *S.* Typhi – and other antigen in the bivalent vaccine) and Shigella flexneri 1b OAg (heterologous from a different species) in PBS and incubated O/N at 4°C with the control (undepleted) reference serum in 1x PBS alone.

### 2.5 Calculations and statistical analysis

ELISA units/mL (EU/mL) are assigned to each tested serum by interpolating from a 5-parameter logistic (5PL) standard curve included on each plate. ELISA units/mL assigned to each sample are the average of the three replicates at the appropriate serum dilution. Validated Excel-based software automatically applies QC criteria for standard curve acceptance (minimum R-square value ≥ 0.96 for the 5PL curve fit to standard dilution series, maximum background < 0.15 OD, minimum value of OD maximum ≥ 3.0, range between 0.5 OD and 2 OD for 1 EU/mL), as well as the control dev <40% to the expected EU/mL both for High and Low control) and variability of samples at each dilution, with EU/mL selected at the sample dilution falling within the linear range of the standard curve.

To calculate the standard curve accuracy, percentage Residual Error RE% [(recalculated value-nominal value)/nominal value*100 (back calculated error)] is plotted in function of the nominal concentration. Lower and upper limit of standard curve accuracy (LLSCA and ULSCA, respectively) were set at the nominal value of the standard curve at the last and the first dilutions, respectively, with a 90% prediction interval of RE% within the acceptance range of [-25%; 25%].

L-SBA analysis was performed using GraphPad 7 Prism (GraphPad Software, La Jolla, CA, USA) by fitting a 4-parameter (4PL) curve of luminescence versus Log sera dilution tested (weighting the data inverse of square luminescence) and constraining the curves to a bottom between 0 and the average of points with maximum bactericidal activity + a standard deviation.

The Lower Limit of Precision and upper limit of precision for ELISA were evaluated for both reproducibility and repeatability by means of a resampling technique. Specifically, the reproducibility (and repeatability) results were randomly resampled 1000 times with replacement and the bounds of 95% bootstrap confidence interval were then used as a proxy of LLP and ULP.

Statistical analyses were performed by Minitab 18 (Minitab Inc., Chicago, IL, USA). ANOVA with variance component analysis (mixed effect model with random factors) was used to estimate the intermediate precision (defined as the variability among different days and different operators), the repeatability (defined as the variability under the same operating conditions over a short interval of time), and to evaluate the contributions of the operator and day of analysis to the variability.

Linearity of ELISA assay was determined by linearity graphs for standard sample, obtained by plotting resulting titers (EU/mL after multiplication per dilution factor) divided by the median of all results (deviation from linearity) vs each dilution with fitted model and 95% CI. Linearity was assessed by 95% CI that must be within the range of 0.7 - 1.3. In addition, log2-transformed GM was plotted against log2-transformed samples dilution.

We applied a normalization technique to the ELISA data for statistical analysis, which allowed us to establish the lower and upper limits of precision (LLP and ULP, respectively). Initially, we normalized the entire batch of ELISA sample data. For accurate low-range measurements, we divided the values at or below 600 by 100. In contrast, to ensure precision in the high-range measurements, we divided values above 600 by 4000. Following normalization, we identified the smallest value in the dataset to set the LLP, representing the assay’s lowest detectable antibody concentration. Conversely, the ULP was determined by detecting the largest value, which reflects the maximum antibody concentration the assay can measure. This approach to normalization guarantees that our assay’s precision is consistent across different levels of antibody concentrations, ensuring dependable results in the detection and quantification of antibodies specific to the *S.* Paratyphi antigen.

Linearity of SBA assay was verified using a linear regression model of Log1.5 transformed dilution values (plotted on x-axis) and Log1.5 transformed of observed mean IC50 values (plotted on y-axis). Relative Accuracy (RA) analysis was also performed using the ratio between the observed mean and nominal mean, where the nominal values have been calculated as the ratio between the previous observed mean and the dilution factor.

For assays specificity the following formula has been used to determine the % of depletion of IgG titer/IC50 (for ELISA and SBA respectively) in the depleted samples compared to the undepleted ones: 100-((signal undepleted-signal depleted)/signal undepleted). Assays have been considered specific if obtaining a depletion of signal >80% or 70% with homologous competitor for ELISA and SBA, respectively and <20 or 30% for ELISA and SBA respectively with the heterologous competitor.

Calculations of LoD and LoQ have been performed accordingly to the ICH guideline Q2(R1) (13), by using the standard deviation (SD) of Log transformed SBA titers obtained for the samples and the lowest serum concentration tested in the assay (here, X=4) according to the following formulas:


LoD= 10^(3.3 ∗ SD)∗X and LoQ= 10^(10 ∗ SD)∗X

## 3 Results

**Figure 1:**
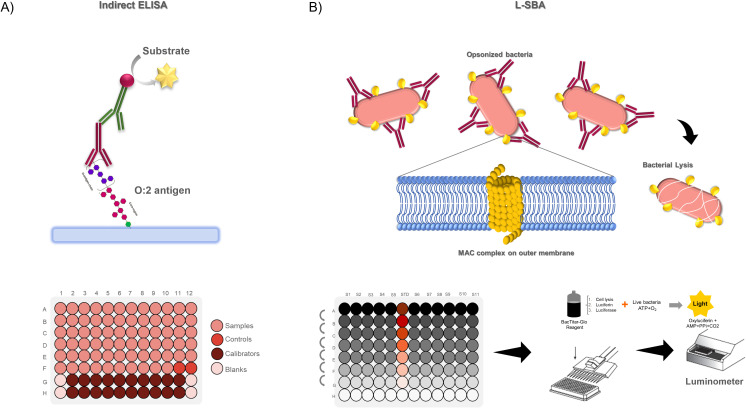
**(A)** Specific anti-O:2 indirect ELISA: OAg is coated to the plate and sera from subjects or standard/controls at appropriate dilutions are added and revealed with anti-human IgG-AP conjugated secondary antibody using pNPP; ELISA layout consists in a standard curve made by 10 calibrators run in duplicate, four blanks, two control samples (High and Low control to validate the plate) and up to 70 properly diluted sample*S.* **(B)** Luminescence serum bactericidal assay measures the ability of an anti-*S.* Paratyphi A antibodies to mediate the formation of the MAC complexes on outer membrane and thus the killing of bacteria present in the inocolum, which is indirectly quantified measuring ATP of live bacteria in wells; A typical L-SBA plates layout consists in up to 11 samples and 1 control sample (to validate the plate) tested in 7 dilution points plus a negative control represented by well with all the reagent but no sera.

To determine the response to a vaccine or after natural exposure in human samples it is critical to quantify the binding of antibodies to the specific target antigen and the ability of those antibodies to induce a functional effect to the target pathogen. To assess the binding of the antibodies, we developed an indirect ELISA method (**Figure 1A**), whereas to assess functionality we developed a serum bactericidal assay against *S.* Paratyphi A, which uses exogenous complement and luminescence as readout (**Figure 1B**). To develop both assays, we generated primary standard sera from high responders and control sera with different reactivity, using serum samples from clinical studies conducted in regions where *S.* Paratyphi A is endemic (4). Results are expressed as EU/mL relative to a calibrated standard curve run in each of the assay for ELISA and as inhibitory concentration 50% in case of SBA, representing the sera dilution able to inhibit 50% of the growth of the bacteria present in the inoculum due to complement mediated killing.

### 3.1 *S.* Paratyphi A anti-O:2 ELISA

**Figure 2:**
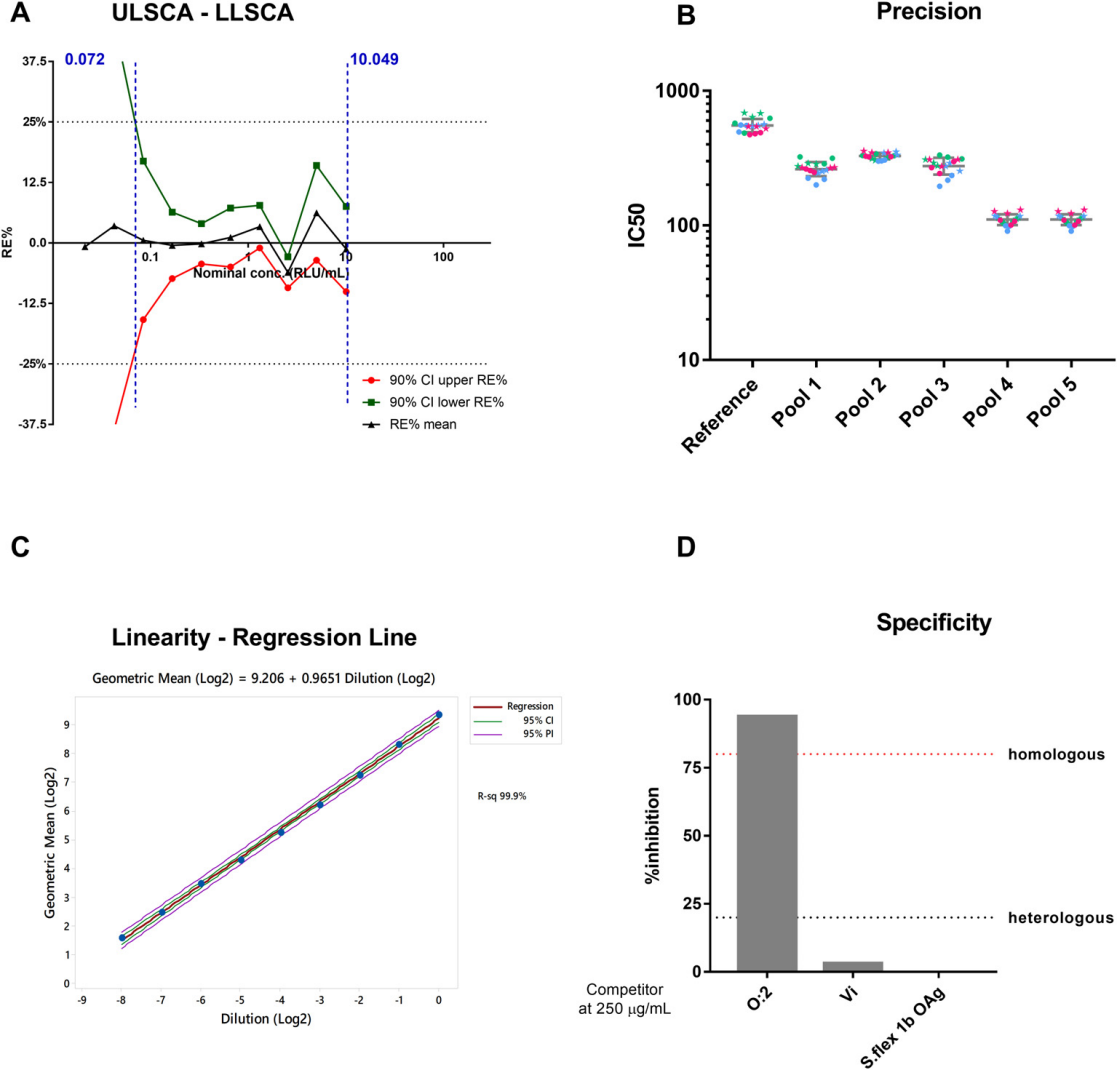
ELISA Assay Parameters **(A)** Upper and Lower Limit Standard Curve Accuracy (ULSCA/LLSCA). Average RE% (black line) and respective upper (red line) and lower (green line) 90% confidence interval for each standard curve point have been plotted in function of nominal concentration. Black horizontal dashed lines represent the acceptance range [+25%; -25%] of RE%. Vertical blue dashed lines represent the values of LLSCA and ULSCA. **(B)** ELISA precision. A total of 18 repeated measurements of EU/mL from single independently handled samples, by two operators on three different day*S.* Single repeats of each operator are represented by star symbols (for operator 1) and circle symbols (for operator 2), repeats on different days are shown in green for day 1, blue for day 2 and pink for day 3. Geometric means and geometric standard deviations from 18 repeats are represented by the grey line for each of the tested sample*S.* **(C)** Linearity. Regression Line. **(D)** Homologous and Heterologous Specificity.

The anti-O:2 reference serum was calibrated to generate a standard curve which is run in each assay and is made by 10 calibrators 2-fold diluted (starting from 10 EU/mL) and two blanks run in duplicate on each plate. Lower and Upper Limits of Standard Curve Accuracy (LLSCA, ULSCA), determined by running 24 times independently the standard curve, resulted to be 0.072 EU/mL and 10.049 EU/mL, respectively (**Figure 2A**). Only values of test samples, run in triplicate and at up to three different dilutions (1:100, 1:400 and 1:160000) in different plates, in which EU/mL felt within the standard accuracy limits have been used to calculate the antigen specific IgG of the sample.

To evaluate the precision of the assay, six samples with different anti-O:2 titers were tested in the ELISA for 3 consecutive days, independently by 2 operators working on the same day*S.* Intermediate precision of tested samples ranged from 2.7% and 9.7%, while the repeatability ranged from 5.6% and 15.8%, well within the expected limits (≤ 25% and ≤ 20%, respectively), thus demonstrating precision of the assay (**Figure 2B**). High (HC) and Low Controls (LC) were also included in the tests at appropriate dilution for a total of 24 repeat*S.* LC resulted in a mean value of 0.42 (0.28-0.57 of CI95%) EU/mL, while high control HC resulted in a mean value of 0.49 (0.38-0.60 of CI95%) EU/mL. Falling within the expected range of HC and LC, which will be run on each plate in a standard assay, will be prerequisite to validate the plate.

To evaluate the linearity, which is the ability of the method to obtain, within a given range, test results which are directly proportional to the concentration of the analyte being measured, the reference serum was prepared as 9 independent dilutions, and tested in a standard ELISA. The analysis of deviation from linearity allowed determination of the Lower Limit of Linearity (LLL) and the Upper Limit of Linearity (ULL), constituting the range of Linearity (0.046 - 7.969 EU/mL/well). The coefficient of determination (R2) of the regression was 0.999, with a slope a 0.965 and 95% confidence interval of the slope, ranging between 0.935 and 0.995 (**Figure 2C**), confirming linearity of the assay.

Lower limit of quantification for the ELISA assay was calculated as the most conservative between lower limit of standard curve accuracy (resulting to be 7.2 EU/mL), lower limit of precision (which resulted to be 13.26 EU/mL) and lower limit of linearity (equal to 4.64 EU/mL), therefore equal to 13.26 EU/mL.

Finally, to assess the ELISA specificity, an initial experiment was performed to determine the lowest concentration of *S.* Paratyphi O:2 able to cause a reduction of the ELISA Units of ≥ 80%. This resulted to be 250 µg/ml, and such concentration was then used to assess heterologous specificity against Vi (the capsular polysaccharide used in the TCV of our bivalent formulation), and against Shigella flexneri 1b OAg (**Figure 2D**). Acceptability criteria were met, as the percentage of inhibition achieved by mixing the samples with the homologous antigen (O:2) was more than 80%, while it was less than 20% when the heterologous antigens were used as competitors (0% and 3.2% for *S.* flex 1b and Vi, respectively). This results confirmed the specificity of the ELISA assay to detect anti- *S.* Paratyphi A OAg IgG.

### 3.2 *S.* Paratyphi A L-SBA

L-SBA assay for *S.* Paratyphi A was developed and optimised by adapting criteria from assays established for clinical testing against other pathogens to the ones previously published (14, 15).

**Figure 3:**
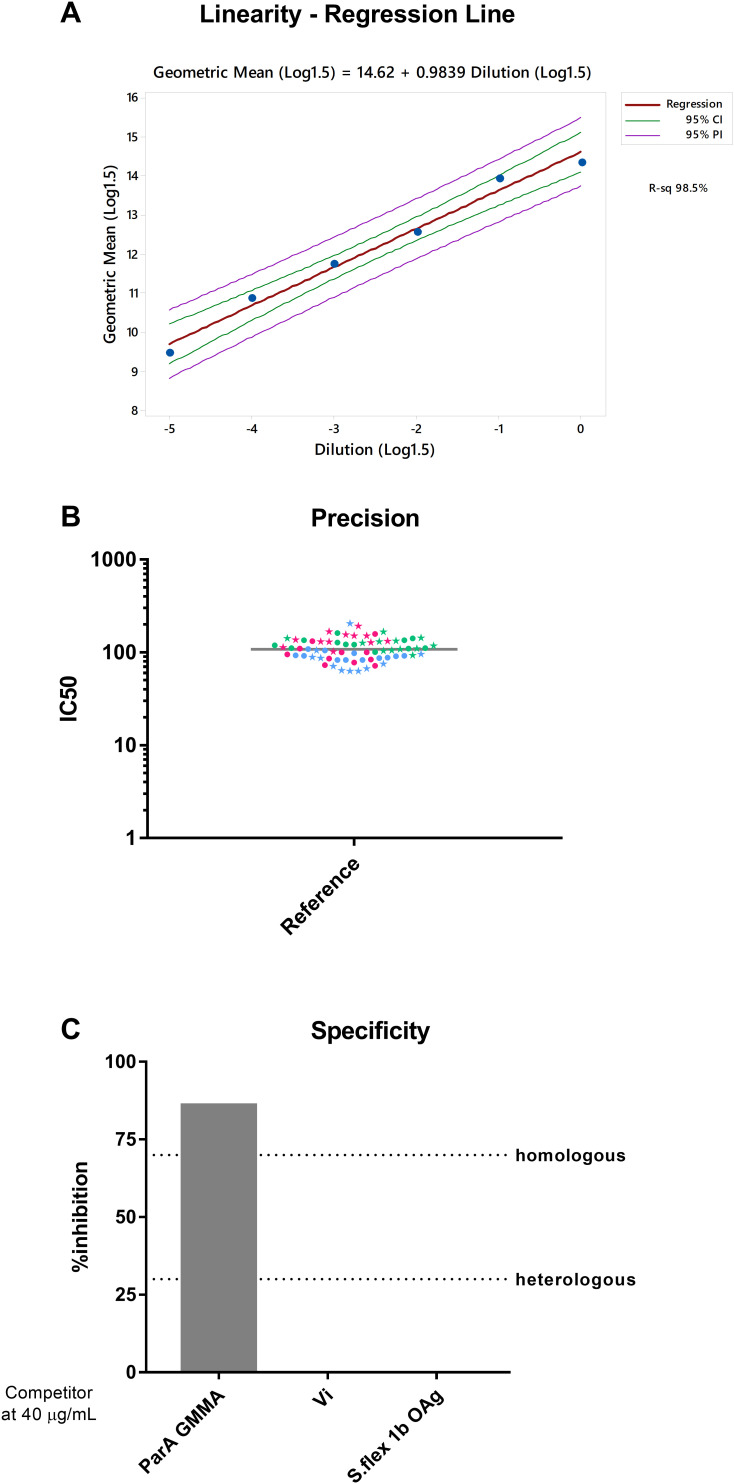
**(A)** Assay linearity: Log1.5 transformed dilution values vs and Log1.5 transformed of observed mean IC50 values **(B)** L-SBA precision. A total of 70 repeated measurements of IC50 from single independently handled reference sample, by two operators on three different day*S.* Single repeats of each operator are represented by star symbols (for operator 1) and circle symbols (for operator 2), repeats on different days are shown in green for day 1, blue for day 2 and pink for day 3. Geometric means and geometric standard deviations from 70 repeats of reference sample are represented by the grey line for each of the tested sample*S.* **(C)** Homologous and Heterologous specificity.

To assess the linearity of the assay, the reference serum was diluted 6 times and run as independent sample in a standard assay. Linear regression analysis (**Figure 3A**) showed a slope of 0.984 (0.816-1.154 95%CI), thus that the linearity condition of the assay is satisfied.

In order to assess the assay precision, the standard serum (**Figure 3B**) was assayed seventy-two times in three different days by two operators and all the IC50 data obtained have been used to determine repeatability and intermediate precision of the assay.

The assay was characterized by a repeatability of 4.4% (LogCV% R) and an intermediate precision of 6.0% (LogCV% IP). The day and the operator were factors that did not influence assay variability (p-values of factors included in the variance component analysis were not statistically significant).

The LoD and LoQ of the assay have been calculated considering the variability of IC50 values obtained for the reference sample obtained in all repeats performed as part of the precision experiments (thus considering also different days and operators, situation with envisaged maximum variability), and resulted in a LoD and LoQ of 7.5 and 27.3 IC50, respectively.

To assess the homologous specificity, GMMA from *S.* Paratyphi A (19) were used to inhibit anti- *S.* Paratyphi A antibody against multiple antigens present in the reference serum. Homologous specificity was determined by calculating the decrease in IC50 observed in SBA when testing reference serum pre-treated with different *S.* Paratyphi A GMMA concentration in comparison to the IC50 obtained by the standard undepleted. GMMA at 40 µg/mL were able to inhibit the homologous signal by 86%, whereas same concentrations of Vi polysaccharide and *S.* flexneri 1b OAg did not show any bactericidal titer inhibition (**Figure 2C**), thus confirming the assay specificity (**Figure 3C**).

### 3.3 Correlation between ELISA IgG titers measured in CHIM study and in GVGH ELISA assays

**Figure 4:**
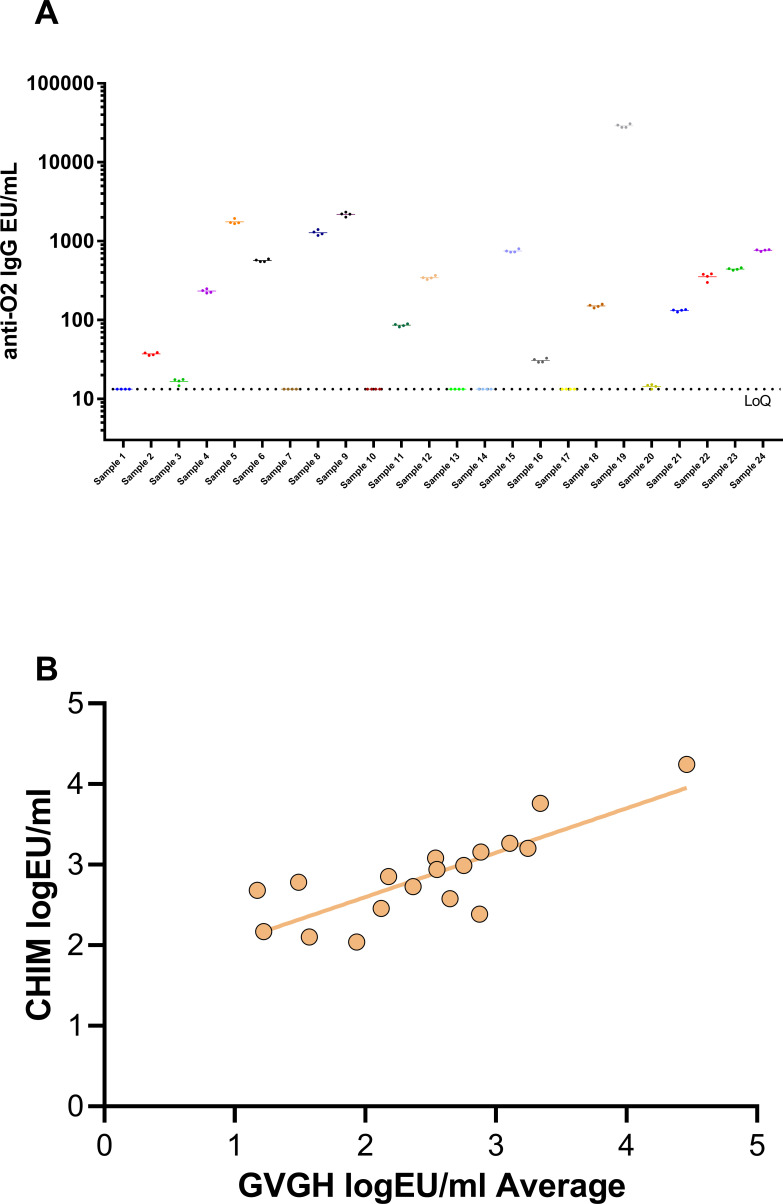
**(A)** 24 samples of CHIM study tested 4 times in ELISA assay by one operator. **(B)** Correlation between GVGH and CHIM ELISA.

Finally, we tested 24 serum samples obtained at different timepoints (0, 28 and 90 days) after infection with a *S.* Paratyphi A strain in CHIM studies) (8, 16). The objectives were to confirm performance of the assays, by testing individual samples multiple times, and to compare the results obtained using different methods developed in different laboratories (GVGH and University of Oxford). Sample were assessed in four independent replicates by one operator in the same day (**Figure 4A**) and the average and standard deviation of the four replicates were used to calculate the CV% among the four measurements, resulting in a CV% ranging from 2.4 and 11%. The geomean of the four results for each sample were used to calculate the correlation with the IgG titers obtained in the original analysis (8) (**Figure 4B**). The Pearson’s Rho was 0.882 with a p-value statistically different from zero with alpha level of 5%, suggesting a good correlation between the two ELISA methods, representing a first effort in obtaining correlation factor between assays performed in different labs and with slightly different methodologies to determine IgG response against O:2.

## 4 Discussion

Despite the successful introduction of vaccines against typhoid fever, the burden of enteric fever is still high and only with the effective introduction of combination vaccines, covering also *S.* Paratyphi A, the control of this disease could become possible. There are different attempts to develop bicomponent vaccines targeting both typhoid and paratyphoid fever and some are under clinical development (11).

Robust, easy to perform and standardized immunoassays are crucial to guarantee high quality evaluation of immune response induced by vaccination, or upon natural exposure. Here we have characterized an ELISA to determine the quantity of IgG antibodies able to bind the purified O:2 antigen, and a L-SBA to evaluate their ability to kill *S.* Paratyphi A. In absence of a correlate of protection against *S.* Paratyphi A, it is important to assess not only the presence of the antigen-specific antibodies, but their functional activity against the target pathogen. We believe that this standardized evaluation of humoral responses will also support studies in which T-cell immunity will be investigated to provide a complete picture of the immune response induced by the vaccine and possibly to identify correlates of protection.

ELISA against *S.* Paratyphi A O:2 demonstrated strong specificity, resulting in >80% signal inhibition with homologous O-Antigen and no inhibition with heterologous Vi polysaccharide or *S.* flexneri 1b O-antigen. The assay linearity was confirmed within the tested range, with repeatability ranging from 2.73 to 8% and reproducibility from 9.06 to 28.38%. Neither the day of assay nor performance by different operators were significantly associated with the overall variability, confirming the assay precision.

The study confirmed that daily fluctuations and different operators do not significantly affect the assay’s variability, indicating high precision. This means the assay consistently produces reliable results, unaffected by external factors like time and operator. Such consistency ensures that the assay accurately measures the intended parameter, reflecting true changes rather than procedural variations.

The LLoQ was 13.26 EU/mL in case of ELISA and 27.3 IC50 for *S.* Paratyphi A, a level that renders them suitable to determine response in clinical trials, accurately.

Both ELISA and SBA methods are designed for high throughput. In fact, 140 and 88 individual samples, for ELISA and SBA respectively, can be assessed by one operator on each day; moreover, the two assays can be easily performed with basic laboratory equipment, allowing a fast transfer to qualified laboratories for clinical testing. Assays miniaturization on 384 well plates can be achieved with minimal protocol adaptation, by using liquid handling automation (20).

Comparison of our assays with others already developed and used to assess clinical samples from a *S.* Paratyphi A CHIM model showed a significant correlation, suggesting robustness and effectiveness of anti-O:2 IgG quantification and validity of comparison of responses obtained by different laboratories and with samples from different trial*S.*

In conclusion, in this work we have extensively characterized the ELISA and L-SBA assays in terms of repeatability, intermediate precision, linearity, specificity, quantification limits and QC acceptance criteria, to make them suitable for testing clinical samples from vaccine trials, or to evaluate natural immunity against *S.* Paratyphi A.

## Data Availability

The original contributions presented in the study are included in the article/supplementary material. Further inquiries can be directed to the corresponding author.
